# Cardiac Myxoma and Cerebrovascular Events: A Retrospective Cohort Study

**DOI:** 10.3389/fneur.2018.00823

**Published:** 2018-10-03

**Authors:** Maria-Ioanna Stefanou, Dominik Rath, Vera Stadler, Hardy Richter, Florian Hennersdorf, Henning F. Lausberg, Mario Lescan, Simon Greulich, Sven Poli, Meinrad P. Gawaz, Ulf Ziemann, Annerose M. Mengel

**Affiliations:** ^1^Department of Neurology and Stroke, and Hertie-Institute for Clinical Brain Research, Eberhard-Karls University of Tübingen, Tübingen, Germany; ^2^Department of Cardiology, Eberhard-Karls University of Tübingen, Tübingen, Germany; ^3^Department of Diagnostic and Interventional Neuroradiology, Eberhard-Karls University of Tübingen, Tübingen, Germany; ^4^Department of Thoracic and Cardiovascular Surgery, Eberhard-Karls University of Tübingen, Tübingen, Germany

**Keywords:** cardiac myxoma, cerebrovascular events, stroke, cerebral ischemia, cardiac surgery

## Abstract

**Background:** Cardiac myxoma (CM) is the most frequent, cardiac benign tumor and is associated with enhanced risk for cerebrovascular events (CVE). Although surgical CM excision is the only curative treatment to prevent CVE recurrence, in recent reports conservative treatment with antiplatelet or anticoagulant agents in high-risk patients with CM-related CVE has been discussed.

**Methods:** Case records at the University Hospital of Tübingen between 2005 and 2017 were screened to identify patients with CM-related CVE. Clinical features, brain and cardiac imaging findings, histological reports, applied treatments and long-term neurological outcomes were assessed.

**Results:** 52 patients with CM were identified and among them, 13 patients with transient ischemic attack, ischemic stroke or retinal ischemia were included to the (to our knowledge) largest reported retrospective study of CM-related CVE. In all identified patients, CVE was the first manifestation of CM; 61% suffered ischemic stroke, 23% transient ischemic attack and 15% retinal ischemia. In 46% of the patients, CVE occurred under antiplatelet or anticoagulation treatment, while 23% of the patients developed recurrent CVE under bridging-antithrombotic-therapy prior to CM surgical excision. Prolonged time interval between CVE and CM-surgery was significantly associated with CVE recurrence (*p* = 0.021). One patient underwent i.v. thrombolysis, followed by thrombectomy, with good post-interventional outcome and no signs of hemorrhagic transformation.

**Discussion:** Our results suggest that antiplatelet or anticoagulation treatment is no alternative to cardiac surgery in patients presenting with CM-related CVE. We found significantly prolonged time-intervals between CVE and CM surgery in patients with recurrent CVE. Therefore, we suggest that the waiting- or bridging-interval with antithrombotic therapy until curative CM excision should be kept as short as possible. Based on our data and review of the literature, we suggest that in patients with CM-related CVE, i.v. thrombolysis and/or endovascular interventions may present safe and efficacious acute treatments.

## Introduction

Cardiac myxoma (CM) is the most frequent primary tumor of the heart, considered a benign, slowly proliferating neoplasm of endocardial origin (1, 2). Due to the low incidence of CM, with approximately 0.5–1 cases per 1,000,000 individuals per year (3), and due to its miscellaneous clinical presentation, with non-specific cardiac, embolic and constitutional symptoms (4), CM identification poses a unique diagnostic challenge in clinical practice.

Among the clinical manifestations of CM, embolic events—due to detached tumor tissue, disseminated thrombotic material overlying the tumor, or a compound of both (5, 6)—have been shown to occur in up to 30–50% of patients (4). Because of the prevalent left-sided location of CM, with 75–90% located in the left atrium of the heart (7), more than 50% of embolic events affect the central nervous system and the retinal arteries (8). CM-related cerebrovascular events (CVE) have been shown to be recurrent (2, 9), typically presenting as embolic ischemic and rarely as hemorrhagic events (10). Although CVE are common among CM patients, population-wise CM comprises a very rare cause of CVE, accounting for merely 0.5% of total CVE cases (11, 12).

Therefore, prospective studies of patients with CM-related CVE are practically unattainable and therapeutic recommendations arise from published case reports or retrospective case series (12–15). Currently, no unequivocal guidelines exist regarding the management of patients with CM-related CVE. Conflicting evidence has been reported about the safety and efficacy of intravenous (i.v.) thrombolysis (15–18). Furthermore, although the only curative treatment to prevent CVE recurrence is surgical CM excision (2, 14), no evidence exists regarding the optimal time-interval between CVE and CM-surgery (19). In view of the CVE recurrence risk in the presence of CM, some authors support emergency CM excision after CVE manifestation and CM diagnosis (19).

However, significant counterarguments to this view have been expressed (10, 14). Firstly, open-heart surgery requires systemic anticoagulation and cardiopulmonary bypass, so that patients with recent preoperative stroke are at increased risk of developing intracranial hemorrhage (20). Thus, some authors have proposed bridging-therapy with antiplatelet or anticoagulation treatment to delay CM surgery (14). Secondly, recent studies assessing the perioperative morbidity and mortality rates, along with the risk for repeated cardiac surgery (i.e., due to mitral valve insufficiency or CM recurrence) after CM-excision have questioned the necessity of operation in cardiopulmonary stable, elderly or high-risk-for-surgery patients (21, 22). Instead, conservative treatment using antiplatelet or anticoagulant agents has been suggested as alternative approach (23). Similarly, conservative treatment has been discussed for patients with tumors less than 2 cm in diameter, as some studies have demonstrated that small CM-size harbors low embolization risk (24).

Considering the clinical uncertainties in regard to management of CM-related CVE, the aim of the present study was to assess the clinical features, applied treatments and clinical outcome in CME-CVE patients. Herein, we present, to our knowledge, the largest retrospective cohort study of patients with CM-related CVE, treated over a 13-year period.

## Methods

**Figure 1:**
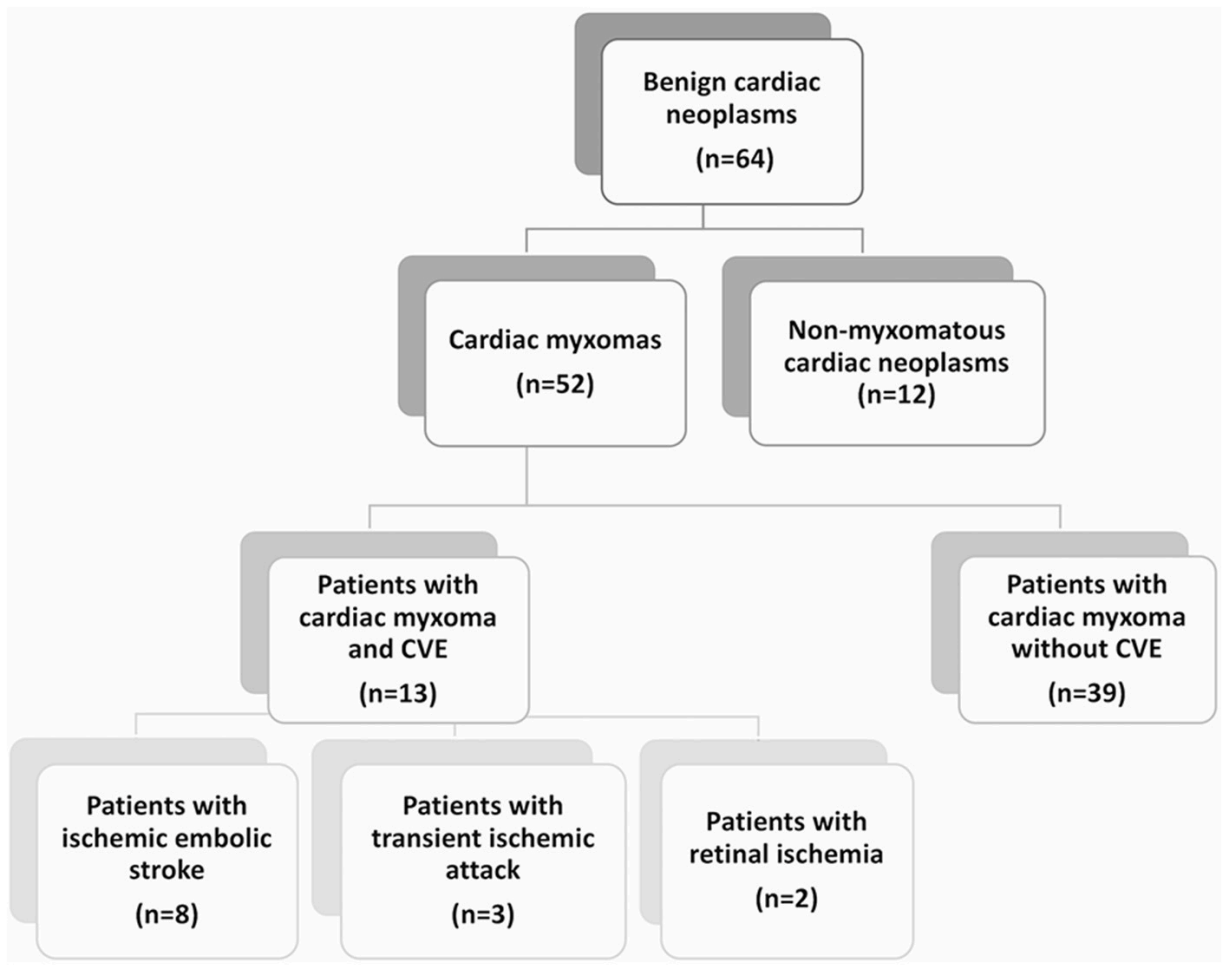
Patient flow chart. The diagram demonstrates the distribution of patients with diagnosed benign cardiac neoplasms of myxomatous and non-myxomatous origin, treated in the University Hospital of Tübingen over a studied period of 13 years. Among them, 13 patients with cardiac myxoma (CM) and cerebrovascular events (CVE) were identified and included in a retrospective cohort study. The majority of the patients (62%) with CM-related CVE presented with ischemic embolic stroke as first CM manifestation, while transient ischemic attacks and retinal ischemic events were noted in 23 and 15% of the patients, respectively.

All patients diagnosed with benign neoplasms of the heart (ICD-10 D15.1), admitted to the University Hospital of Tübingen between 2005 and 2017, were identified based on an electronic database-search. The medical records, cardiac imaging findings and histological reports were reviewed and patients with non-myxomatous benign cardiac neoplasms were excluded (Figure 1). From this collective, patients with concomitant diagnosis of CVE (ICD-10 I60-I69 or G45), based on the clinical and brain imaging findings, were identified. The case records of these patients were further reviewed in regard to clinical manifestations, brain and cardiac imaging findings, histological reports and applied treatments. Outcome assessment was performed at the end of the study period via a structured telephone interview assessing the modified Rankin Scale (mRS) and history of cardiovascular events. Good outcome corresponded to mRS scores of 0–2, and poor outcomes included death or dependency (mRS scores of 3–5). 10/13 patients were included and 3 were lost in follow-up. The study was approved by the institutional ethics committee (Protocol number 379/2018B02).

Baseline characteristics of CM-patients with vs. without CVE are presented in Table 1. For continuous data means ± standard deviation (SD) are reported. Categorical variables are summarized by counts and percentages. To assess whether association of CVE occurrence with age, sex, CM size or cardiovascular risk factors existed in CM-patients with vs. without CVE, chi-square tests were performed. To compare the time elapsed between CVE and CM surgical excision in patients with singular vs. recurrent CVE, Mann-Whitney U Test was used. To exclude that observed differences in respect to concomitant cardiovascular risk factors or CM size existed between patients with singular vs. recurrent CVE, chi-square tests were performed. The significance level was determined at *p* < 0.05. All statistical analyses were computed with SPSS 22.0 (IBM, Chicago, IL).

**Table 1:** Baseline characteristics of patients with cardiac myxoma with CVE vs. without CVE.

**Characteristics**	**Patients with CVE (*n* = 13)**	**Patients without CVE (*n* = 39)**	***p-*values**
Age [mean (±SD)]	61.7 (±17.5)	63.6 (±12.5)	0.308
**SEX**			
Female [*n* (%)]	8 (62%)	24 (61%)	1.000
Male [*n* (%)]	5 (38%)	15 (38%)	1.000
**CARDIAC MYXOMA**			
Average size (cm^2^) [mean (±SD)]	6.3 (± 5.6)	9.6 (± 7.7)	0.097
Location in LA [n (%)]	13 (100%)	33 (85%)^§^	0.133
**CARDIOVASCULAR RISK FACTORS**			
Hypertension [*n* (%)]	10 (76%)	25 (64%)	0.393
Smoking [*n* (%)]	2 (15%)	8 (20%)	0.685
Atrial fibrillation [*n* (%)]	2 (15%)	5 (12%)	0.815
Diabetes mellitus [*n* (%)]	3 (23%)	4 (10%)	0.241
Hyperlipidemia [n (%)]	1 (7%)	6 (15%)	0.482
Coronary heart disease [n (%)]	4 (30%)	10 (25%)	0.718

§ *In the remaining 6 (15%) patients CM was located in the right atrium. CVE, cerebrovascular event; LA, left atrium*.

## Results

### Baseline characteristics and descriptive statistics of patients with CM-related CVE

A total of 64 patients with diagnosis of benign cardiac tumors were identified. Of those, 12 patients diagnosed with non-myxomatous tumors (10 with papillary fibroelastoma and 2 with cardiac lipoma) were excluded (Figure 1). Of the remaining 52 patients, 51 underwent surgery and the diagnosis of CM was histologically verified. Among them, 12 patients with CVE and histologically verified CM and 1 patient with CVE and suspected CM upon cardiac imaging were identified. Accordingly, the CM-CVE group consisted of 13 patients (25% of the CM patients), 8 women (62%) and 5 men (38%), with mean age 61.7 ± 17.5 years. Concomitant cardiovascular risk factors were common among CVE patients (76%). However, no statistically significant differences in respect to age, sex, CM size or cardiovascular risk factors existed in CM-patients with vs. without CVE (Table 1).

### Clinical presentation of patients with CM-related CVE

In all cases, CVE was the first manifestation of CM, which led to CM diagnosis. None of the 13 patients showed further cardiac (i.e., dyspnoea, palpitations, syncope) or constitutional (i.e., fever, asthenia, weight loss) symptoms. One patient (0.7%) presented with concomitant peripheral embolization to the right upper extremity. Of the 13 patients, 6 (46%) had a positive history for CVE-suggestive symptoms.

**Figure 2:**
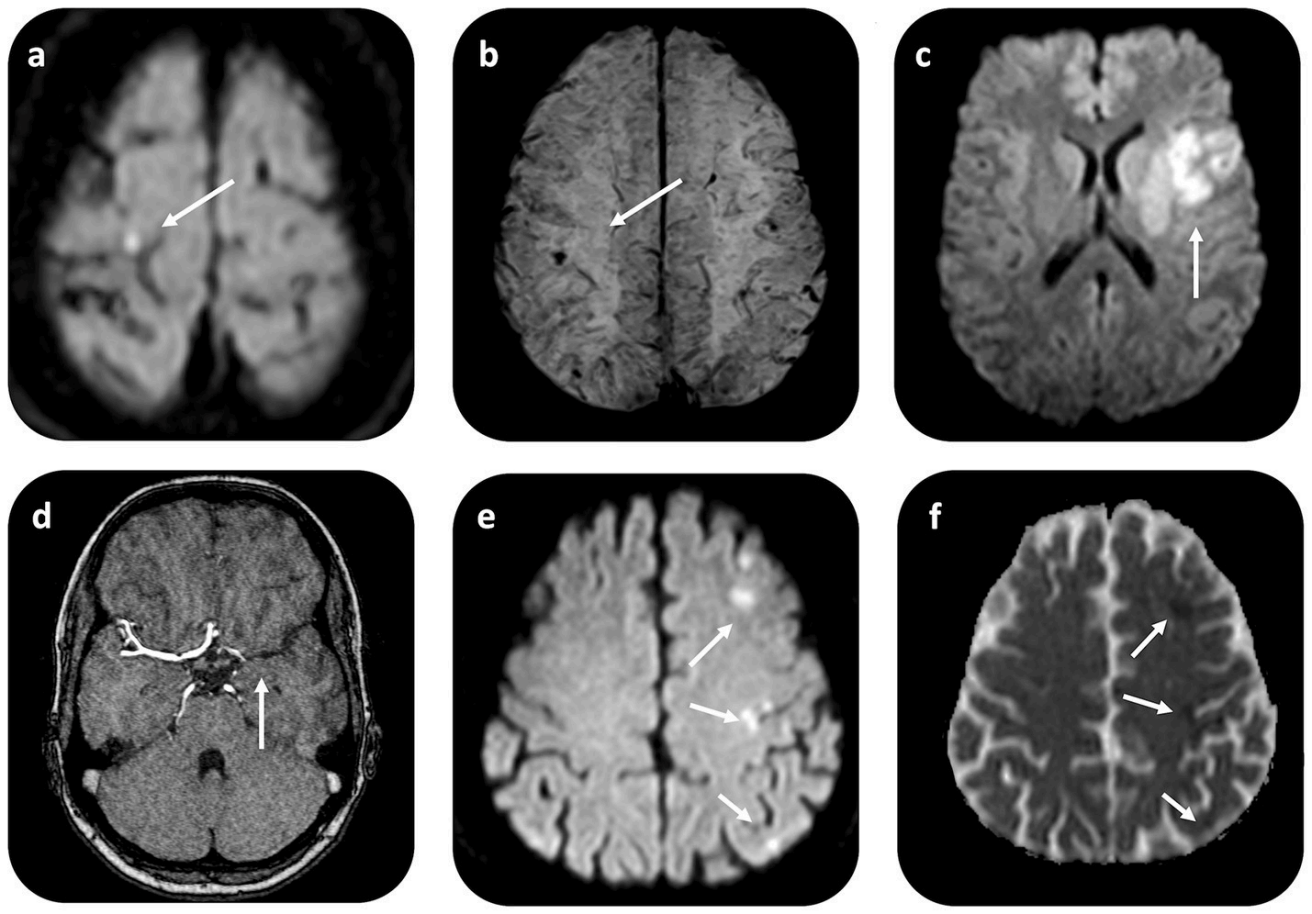
Brain MRI in patients with CM-related CVE. Axial diffusion weighted (DWI) MR imaging in a patient with CM-related CVE **(a)** shows restricted diffusion with high signal on DWI (*b* = 1,000 mm/s^2^) and low signal intensity on apparent diffusion coefficient (ADC) map (not shown) in the right precentral gyrus. On gradient recalled echo (GRE) T2*-weighted imaging **(b)** of the same patient, punctuate low signal intensity compatible with hemorrhagic transformation i.e., microbleed within the cortical infarct area is noted. In another patient with CM-related stroke, who underwent i.v. rtPA administration and thrombectomy, axial DWI **(c)** shows restricted diffusion in the left MCA territory, due to proximal left-MCA occlusion shown on MRAngiography **(d)**. An illustrative case of another patient with CM-related CVE with scattered-brain infarct pattern, demonstrated as multiple foci of restricted diffusion on DWI **(e)** imaging, with corresponding ADC restriction (arrows) **(f)**. CVE, cardiovascular event; CM, cardiac myxoma.

Regarding the type of CVE, in the majority of the patients (*n* = 8, 62%) ischemic embolic stroke was the first CM manifestation. Furthermore, 3 patients (23%) presented with transient ischemic attack (TIA) and 2 patients (15%) with retinal ischemia. Only 1 patient (0.7%) was diagnosed with ischemic stroke with secondary, partial hemorrhagic transformation; yet, the extent of the intracerebral hemorrhage was limited to isolated cortical hemorrhagic lesions (Figure 2). No cases of primary intracerebral or subarachnoidal hemorrhage were reported. In the acute phase, patients with ischemic stroke presented with relatively mild clinical deficits, as reflected in the NIHSS scores (2.7 ± 2.9), with the exception of 1 patient, who presented with a middle cerebral artery (MCA) syndrome (and NIHSS 10).

### Brain and cardiac imaging findings in patients with CM-related CVE

Brain computed tomography (CT) and/or magnetic resonance imaging (MRI) scans were available for all patients (only CT available in *n* = 5, CT and MRI available in *n* = 8 patients). In 10 patients (76%), abnormal imaging findings were recorded. These were compatible with acute (*n* = 6, 46% of the CM-CVE population) or subacute/chronic (*n* = 4, 30% of the CM-CVE population) ischemic infarcts of suspected embolic etiology. Scattered brain infarctions on MRI, often affecting several vascular territories, were common (*n* = 5, 71%) among patients (Figures 2a,b), who presented with acute cerebral ischemia. CT and/or MR angiography were also available for all patients. No intracranial cerebral aneurysms or pseudoaneurysms were reported.

**Figure 3:**
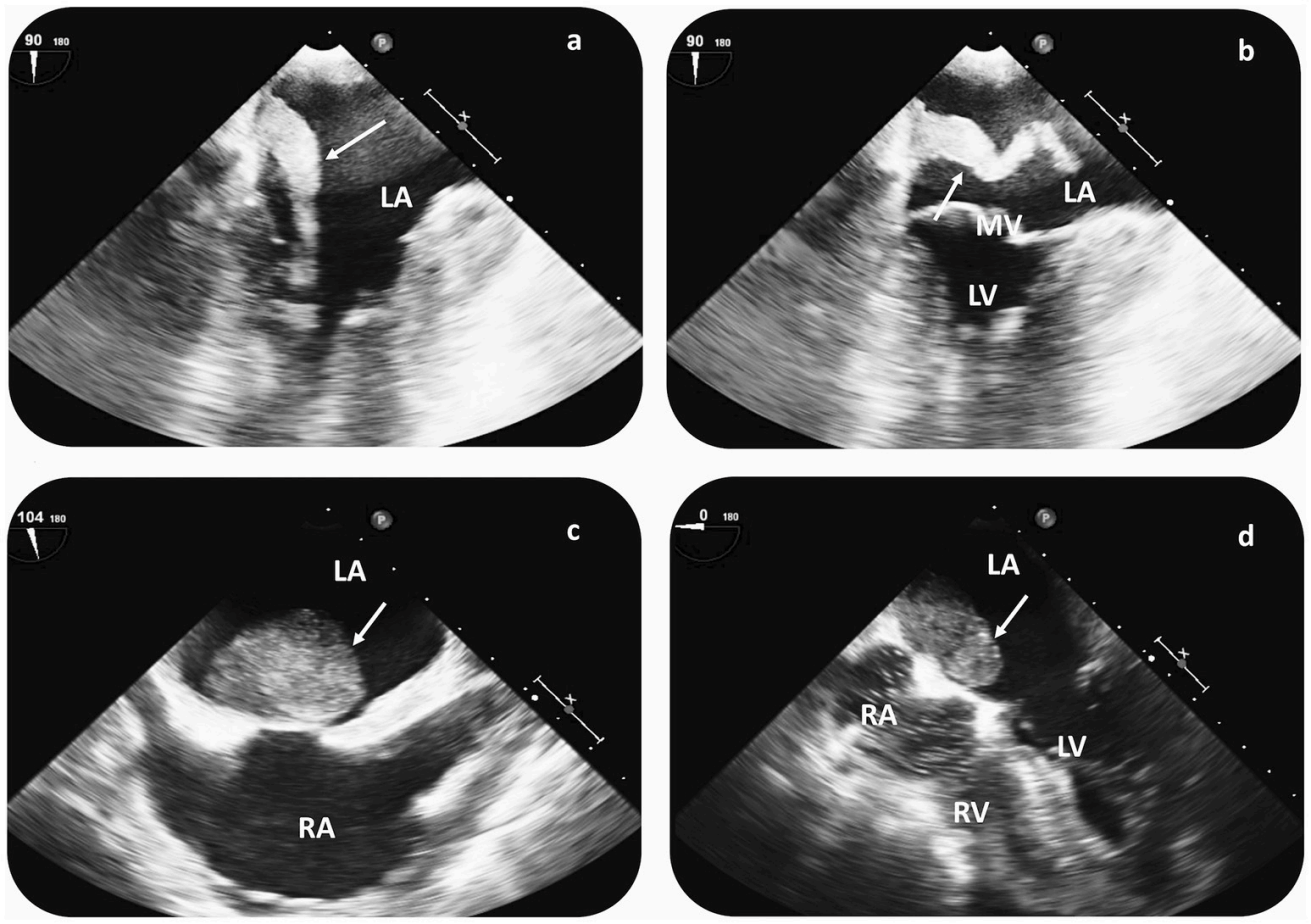
Cardiac imaging findings in patients with CM-related CVE prior to surgical resection. Transesophageal echocardiography (TEE) in a patient with recurrent CVE demonstrates a hypermobile structure, compatible with atrial myxoma (arrow) in the LA **(a,b)**. TEE reveals a large LA myxoma (24 × 34 mm) with a solid appearance in a second patient with singular CVE **(c,d)**. In both cases, CM diagnosis was histologically verified after surgical resection. CVE, cerebrovascular events; LA, left atrium; RA, right atrium; LV, left ventricle; RV, right ventricle; MV, mitral valve.

Transthoracic echocardiogram (TTE) was part of the diagnostic work-up in all CVE patients; yet, additional transesophageal echocardiography (TEE) was performed in all cases, upon CM suspicion (Figure 3). Cardiac CT and MRI scans were available in 1 and 3 patients, respectively. Average (TEE-demonstrated) CM size was 6.3 ± 5.6 cm^2^ (median 4.4 cm^2^), while in all patients CM were localized in the left atrium (LA). All but 1 patient (aged 21 without concomitant cardiovascular risk factors) underwent coronary angiography to assess for concomitant coronary heart disease prior to cardiac surgery. In 10 patients (76%), CM operation was delayed until coronary angiography could be performed. In 2 patients (16%), significant coronary artery stenosis was diagnosed, while 3 patients (23%) had three-vessel coronary artery disease, but without coronary stenoses requiring angioplasty.

### Therapeutic management and clinical outcome of patients with CM-related CVE

#### Intravenous thrombolysis and intra-arterial thrombectomy for acute CM-related ischemic stroke

In the acute phase of ischemic stroke, only 1 patient, who presented within the time-window for i.v. thrombolysis with an MCA syndrome due to proximal MCA occlusion (and NIHSS 10), underwent i.v. recombinant tissue plasminogen activator (rtPA) administration. Furthermore, in this patient, intra-arterial thrombectomy was performed achieving full recanalization of the occluded vessel. No complications and particularly, no post-interventional hemorrhagic conversion were noted. According to the file records, the patient's neurological deficits resolved almost completely over the following months (NHSS 1 after 3 months).

We reviewed the reasons why i.v. thrombolysis was not performed in the rest of the patients, who presented with CM-related ischemic stroke. In 1 patient, with prominent peripheral embolization to the right upper extremity and laboratory findings of elevated inflammatory markers, rtPA administration was withheld due to initial suspicion of endocarditis. Because of the mild clinical deficits (NIHSS 2 upon admission) and the absence of cerebral artery occlusion no endovascular intervention was required. In another patient, rtPA administration was withheld upon imaging findings of cortical microbleeds within the infarcted area upon MR imaging (Figures 2a,b). The remaining 7 patients with ischemic stroke presented with minor stroke (NIHSS < 4) outside the time window for i.v. thrombolysis.

#### Pharmacotherapy is no alternative to surgical treatment and bridging-therapy with antiplatelet or anticoagulant agents does not prevent CVE recurrence

Regarding the pharmacological pre-treatments, CVE occurred under antiplatelet or anticoagulation treatment in almost half (46%) of the CM-CVE patients. More precisely, 3 patients developed CVE under antiplatelet (aspirin, *n* = 2; clopidogrel, *n* = 1) and 3 patients under anticoagulation treatments (warfarin, *n* = 2, for one of these patients INR was documented at the time of CVE and was 3.0; dabigatran, *n* = 1, with dabigatran level 175 ng/ml at the time of CVE).

After CVE diagnosis and until cardiac surgery, most patients received bridging-therapy with antiplatelet (aspirin, *n* = 3 patients) or anticoagulant (low molecular weight heparin, *n* = 3; heparin, *n* = 3; dabigatran, *n* = 1; apixaban, *n* = 1; warfarin, *n* = 1) regimens. However, 1 patient developed recurrent CVE under treatment with dabigatran (150 mg PO b.i.d.), 1 patient under sufficient warfarin therapy (INR 3.0), and 1 patient under aspirin (100 mg PO q.d.). Neither antiplatelet nor anticoagulation therapy prevented CVE recurrence in 23% of the patients.

#### Surgical resection of cardiac myxoma and clinical outcome in operated patients

All but 1 patient, who denied surgery, underwent CM surgical excision. Isolated transseptal CM resection was performed in 6 patients, CM resection with reconstruction of atrial septum with autologous pericardial patch in 4 patients (in the same session, in 1 patient a left anterior descending (LAD) artery bypass and in another patient a superior vena cava thrombectomy were performed), and CM resection and reconstruction of mitral valve in 2 patients (in 1 of them, in the same session with LAD artery bypass).

Out of the 12 operated patients, 9 presented no post-operative complications, 1 developed sick sinus syndrome with atrioventricular block, while another patient developed a deep wound infection. Crucially, none of the patients—particularly, none of the “early operated” patients (14.6 ± 12.9 days) with ischemic infarcts—developed intracerebral hemorrhagic conversion of the ischemic infarcts. 1 patient presented with recurrent myxoma, with secondary manifestation (possibly dissemination after the LA-CM surgery) in the left ventricle. This patient developed recurrent CVE under warfarin therapy, so that revision of the surgery with *in toto* CM excision was performed, 2 years later. After the second surgery, no CM or CVE recurrence were reported. In the remaining 11 operated patients, no CM or CVE recurrence was reported after CM excision at the end of study period (follow-up duration 4.2 ± 3.6 years). Poor long-term outcome (death due to other etiology) was documented in 1 case (7%), while in the remaining 93% of patients good long-term outcome (corresponding to mRS scores 0–2) was reported. After CM surgical removal, 6 patients were treated with aspirin (100 mg PO q.d.), 1 with warfarin therapy for 6 months due to intra-operatively detected vena cava superior thrombosis and 1 with rivaroxaban (20 mg PO q.d.) due to atrial fibrillation.

We further reviewed the reasons for delaying CM surgery. In the majority of the patients (*n* = 10) surgery was delayed until coronary angiography could be performed; yet, in 1 patient with retinal ischemia CM surgery was significantly delayed due to initially misdiagnosed CVE-etiology. The second (contralateral) retinal ischemia led to more detailed diagnostic work-up including TEE and led to CM diagnosis.

In all patients, who underwent surgery, histological data—confirming CM diagnosis—were available. Importantly, no chronic organized thrombi overlaying the tumors were documented in the pathological reports, while in none of the patients, atypical cells were reported.

#### Risk of CVE recurrence increases with time elapsing between CVE and CM surgical resection

**Figure 4:**
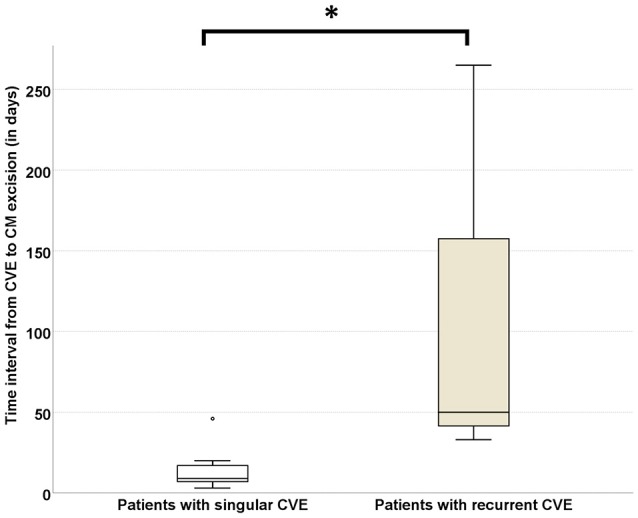
Risk of CVE recurrence increases with time elapsing between CVE and CM surgical resection. The time interval from CVE to CM surgical resection was significantly longer in patients with recurrent (*n* = 3) CVE (116 ± 129.31 days) compared to patients with singular (*n* = 10) CVE (14.6 ± 12.97 days), (*U* = 1, *p* = 0.021). CVE, cardiovascular event; CM, cardiac myxoma (Significance level < 0.05 is denoted with *).

As CVE recurrence was noted—despite bridging-therapy—in 23% of the CM-CVE patients, we compared the elapsed time between CVE and CM surgical excision in the patient groups with singular (*n* = 10) vs. recurrent (*n* = 3) CVE. Our results indicate that the time interval from CVE to CM surgical resection was significantly longer in patients with recurrent CVE (116 ± 129.31 days) compared to patients with singular CVE (14.6 ± 12.9 days), (*U* = 1, *p* = 0.021) (Figure 4).

#### Risk of CVE recurrence is independent from concomitant cardiovascular risk factors and size of cardiac myxoma

**Figure 5:**
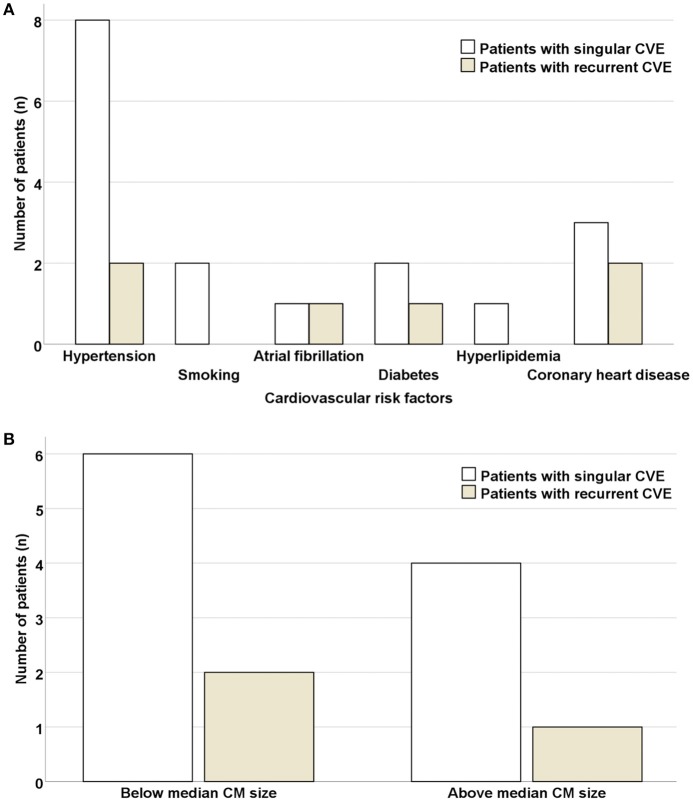
Risk of CVE recurrence is independent of concomitant cardiovascular risk factors and size of cardiac myxoma. No significant differences were noted in respect to concomitant cardiovascular risk factors **(A)** or CM size (allocating patients in 2 categories, above or below the CM median size = 4.4 cm^2^) **(B)** in patients with singular vs. recurrent CVE. CVE, cardiovascular event; CM, cardiac myxoma.

No significant differences in respect to concomitant cardiovascular risk factors (hypertension, *p* = 0.631; smoking, *p* = 0.400; atrial fibrillation, *p* = 0.326; diabetes, *p* = 0.631; hyperlipidemia, *p* = 0.569; coronary heart disease, *p* = 0.252) were noted in patients with singular vs. recurrent CVE (Figure 5). Similarly, no significant differences regarding CM size (allocating patients in 2 categories, above or below the CM median size = 4.4 cm^2^) were noted in patients with singular vs. recurrent CVE (*p* = 0.835). Furthermore, 24% of patients had CM size under 2 cm^2^.

## Discussion

In this study we present, to our knowledge, the largest cohort of patients with CM-related CVE, diagnosed and treated over a 13-year period. Overall, neurological manifestations compatible with CVE affected 25% of CM patients. This finding is in line with previous reports on incidence of neurological manifestations in the presence of CM (2, 25). Furthermore, the noted 1.6:1 female-to-male ratio is in accordance with the reported female predominance of CM, with ratios varying from 2:1 to 3:1 (2, 7, 26). In our analysis, the mean age of patients with CM-related CVE was 61.7 ± 17.5 years. According to larger CM cohort studies, however, CM occur in all age-groups and are particularly frequent from the third to sixth decades of life (2, 10). A plausible explanation for the slightly advanced age of our CM-CVE cohort could be attributed to concomitant cardiovascular risk factors, which were prevalent among patients (76%).

Regarding the clinical presentation of CM-related CVE, ischemic embolic stroke was prevalent as first CM manifestation (affecting 61% of patients), followed by TIA (23%) and retinal ischemia (15%). Although cardiac (i.e., symptoms of cardiac failure or obstruction), constitutional (i.e., fever, asthenia, weight loss) and peripheral embolic symptoms are common among CM patients (2, 25), only one patient with CM-related CVE in our study had reported peripheral embolization to the right upper extremity at the time of CVE. The low prevalence of concomitant systemic manifestations in our cohort may be attributed to the retrospective study design. In particular, as the study was commenced in a tertiary referral hospital, subtle and non-specific clinical symptoms, which did not require hospitalization, may have escaped mention in the clinical reports.

**Figure 6:**
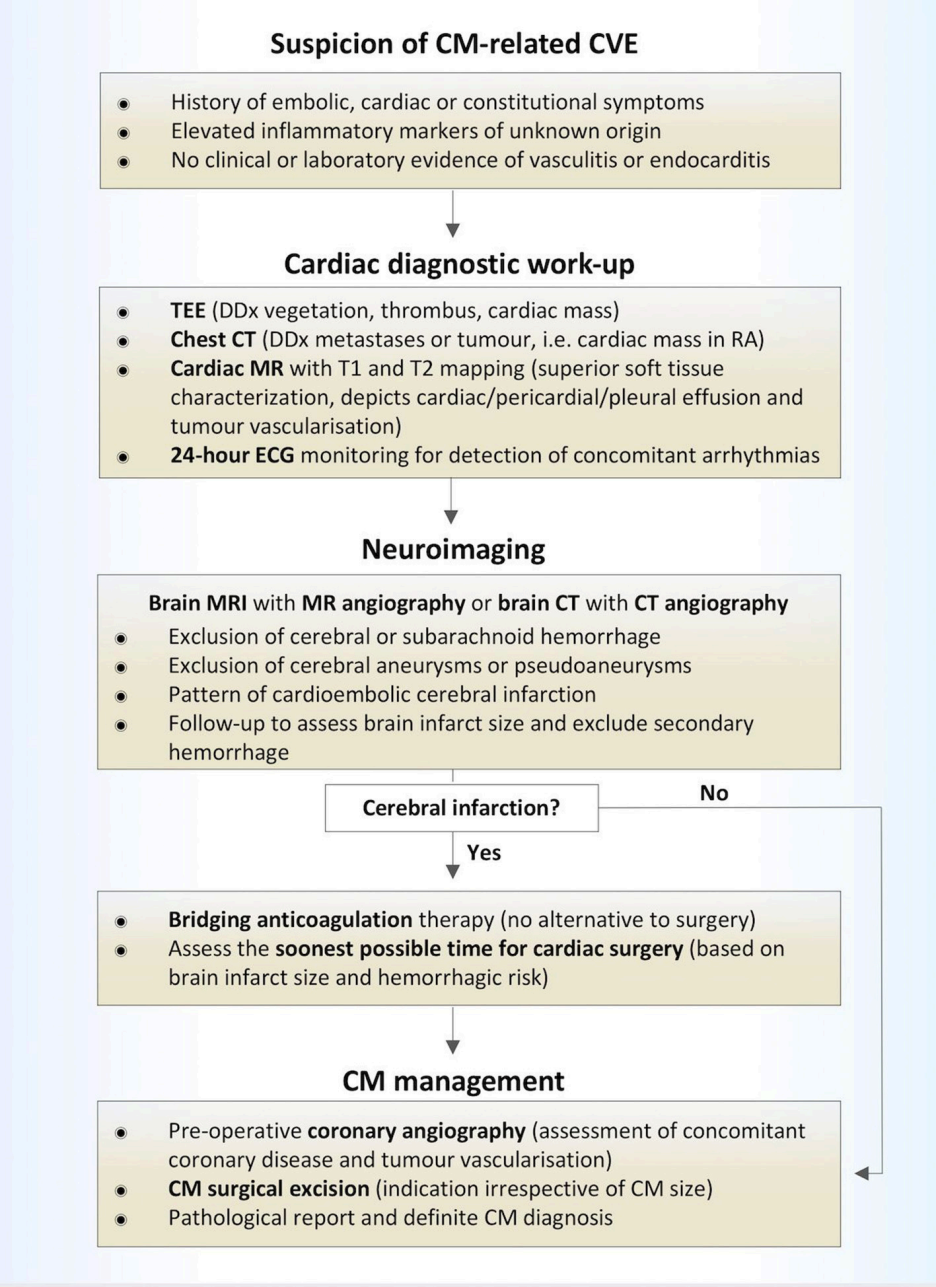
Diagnostic and treatment algorithm of patients with CM-related CVE. CVE, cardiovascular event; CM, cardiac myxoma; TEE, transesophageal echocardiography; DDx, differential diagnosis; CT, computed tomorgraphy; MR, magnetic resonance imaging; RA, right atrium; ECG, electrocardiography.

At the time of CVE diagnosis, 2 patients (15%), presented with abnormal laboratory findings and elevated inflammatory markers and were initially misdiagnosed. On one occasion, in the aforementioned patient with peripheral embolization to the right upper extremity, endocarditis was suspected. In view of the high-risk for post-thrombolytic intracerebral hemorrhage in acute stroke in the setting of endocarditis (27), no i.v. thrombolysis was administered to this patient. On a second occasion, one patient with retinal artery occlusion and elevated inflammatory markers was initially misdiagnosed with temporal arteritis and received treatment with aspirin and oral corticosteroids, during which she developed recurrent retinal ischemia in the other eye. These illustrative cases indicate, that as the clinical presentation of CM is miscellaneous, CM often remains undiagnosed upon first CVE manifestation. Nevertheless, as abnormalities in serologic and hematologic investigations are not uncommon, but present in almost one third of CM patients (2, 7, 26), high clinical awareness is warranted to instigate promptly cardiac imaging, preferably with transesophageal echocardiography, in patients presenting with embolic CVE and elevated inflammatory markers of unknown origin (28). While chest CT holds a crucial role in the diagnostic work-up of cardiac masses and particularly of those located in the right atrium, cardiac MR imaging with T1 and T2 mapping sequences has been established as a superior imaging modality with respect to soft tissue characterization compared to echocardiography or CT alone (29). Furthermore, cardiac MR can reveal cardiac, pericardiac or pleural effusion or prominent tumor vascularisation, suggestive of malignancy (30). Therefore, we recommend early consideration of chest CT or preferably cardiac MR upon CM suspicion (Figure 6).

Predominant patterns on neuroimaging among CM patients, presenting with acute CVE, were scattered brain infarctions, often affecting several vascular territories on MRI (in 71% of patients). These findings may correlate with the “mild” clinical phenotype of CM-related CVE, with average NIHS scores of 2.7 ± 2.9 upon admission (31). Contrary to published reports (14, 19), no patient was diagnosed with primary intracerebral or subarachnoid hemorrhage in our cohort. Moreover, no intracerebral aneurysms or pseudoaneurysms were detected upon CT- or MR-angiography. Yet, in one patient cortical hemorrhagic transformation within the ischemic lesion was shown.

Intracerebral hemorrhage in the setting of CM has been suggested to occur as detached tumor material congregates into small brain arteries, causing vessel-wall destruction (14). The same mechanism may lead to vessel-wall infiltration and pseudo-/aneurysm formation (14, 32). Moreover, rare cases of malignant transformation of CM tissue, invading the cerebral arteries and growing within the brain parenchyma, have been reported (33, 34). In our analysis none of the patients presented with suspicious intracerebral masses, while the histological studies of cardiac tissue revealed no atypical cells or signs of malignancy. However, in light of the previous evidence, which supports an increased risk of intracranial aneurysmatic or myxomatous formations in association with CM, we recommend that brain imaging should be considered in all CM patients prior to cardiac surgery.

Regarding the acute treatment of CM-related CVE, previously published case reports have discussed the safety and efficacy of i.v. thrombolysis (15–17, 35, 36). In a recent review of 16 published cases of CM patients with acute stroke, who received i.v. thrombolysis (18), an incidence of cerebral hemorrhage in 5 out of 16 patients (31%) was reported—a ratio slighly higher than the one reported in the ECASS III study (27%) (37). However, only cases of small intracranial hemorrhage or microbleeding after i.v. thrombolysis were reported (18). In these 5 cases, no major clinical deterioration followed the bleeding events. Furthermore, the reported CM-patients, who suffered intracranial hemorrhage after i.v. thrombolysis were significantly older (with an increased comparative risk of bleeding after i.v. thrombolysis) than the patient group of the ECASS III study (18, 37). No hemorrhage was reported in patients, who were treated with intraarterial thrombolysis or thrombectomy (18, 38–40). In particular, endovascular procedures i.e., intra-arterial thrombolysis and thrombectomy have been proposed to minimize the risk of hemorrhage in patients with known CM, by firstly, excluding (pseudo)aneurysm with diagnostic angiography and, secondly, minimizing fibrinolytic exposure to unaffected vessels (38, 39). In our study, one patient with MCA occlusion received i.v. thrombolysis, followed by thrombectomy, with good post-intervantional outcome and no signs of hemorrhagic transformation.

In practice, the presence of CM in most patients, that are acutely admitted with CVE is not known. Therefore, only if endocarditis is highly suspected, withholding thrombolysis may be critically discussed (27). Yet, if CM is already diagnosed and the patient is admitted with acute CVE, we recommend performance of cerebral angiography prior to rtPA administration for bleeding risk stratification due to occult tumor emboli or aneurysms in CM patients (17). Accordingly, thrombectomy should be early discussed as alternative or add-on therapy to i.v. thrombolysis. Crucially, as the rates of hemorrhagic transformation in reported CM-patients, who received thrombolysis for acute CVE approximate the rates of intracerebral hemorrhage in the general population after i.v. rtPA thrombolysis (18, 37), we suggest that thrombolysis should be considered, after exclusion of intracerebral aneurysms.

Furthermore, our data show that pharmacotherapy with antiplatelet or anticoagulation regimens is no alternative to CM surgical excision, as CVE occurred under antiplatelet or anticoagulation treatment in almost half (46%) of the CM-CVE patients. Additionally, since CVE recurrence in three (23%) of our patients was observed under bridging-antithrombotic-therapy, the bridging-interval from CVE diagnosis to CM surgery should be kept to a minimum. After CM diagnosis, diagnostic coronary angiography should be performed to assess for concomitant coronary artery disease prior to surgery, without prolonging the waiting interval to CM excision.

Due to the small sample size and rarity of CM the present study is statistically underpowered. Yet, our results suggest that the risk of CVE recurrence increases with the time elapsing between CVE and CM surgical excision, as significantly longer time interval from CVE to cardiac surgery was found in three patients with recurrent compared to ten patients with singular CVE. Moreover, the recurrence risk appeared to be independent of concomitant cardiovascular risk factors and independent of CM size (41). In respect to the latter, our findings are in line with studies, suggesting that not CM size, but tumor friability is related to embolic risk (41). As 24% of patients with CM-related CVE in this cohort had CM size under 2 cm^2^, our findings are in contrast to reports of other authors (24), who have supported that tumors less than 2 cm in diameter harbor low embolization risk. Finally, in our study, no intracerebral hemorrhage was noted in the “early” operated patient group (14.6 ± 12.9 days). Taken together, these results indicate that early surgery should be considered in patients with TIA or retinal ischemia (without acute intracerebral infarcts), whereas in patients with cerebral infarcts, early operation should be discussed interdisciplinary and evaluated on individual basis (20).

Some of the limitations of the present analysis involve, apart from the inevitably small number of included patients and the consequently low statistical power of the study, the retrospective nature of the study and the use of ICD codes. Furthermore, the fact that only one identified patient received i.v. thrombolysis allows cautious inferences regarding the efficacy and safety of i.v. rtPA administration. In line with the cited literature, we recommend individual risk-benefit assessment, in view of individual neurological deficits and concomitant risk factors for bleeding (i.e., arterial hypertension, hematological status) for all patients presenting with CM-related CVE within the time-window for thrombolytic treatments.

In conclusion, we report clinical features, applied treatment and clinical outcome from a case-cohort of 13 consecutive patients with CM-related CVE. Based on our results, we suggest that, as antiplatelet or anticoagulation treatment is no alternative to CM surgery, the bridging- interval to curative CM excision should be kept as short as possible. This recommendation is supported by the findings of significantly prolonged time intervals between CVE and CM surgery in patients with recurrent CM-related CVE. We provide evidence that embolic ischemic strokes due to CM commonly present upon imaging with multi-infarct scattered patterns of ischemia, which may explain the frequently noted “mild” clinical phenotype of CM-CVE. However, in patients with CM-related CVE, who present with relevant neurological deficits within the time-window for thrombolytic treatments, on condition that cerebral aneurysms and pseudoaneurysms have been excluded, i.v. thrombolysis and/or endovascular interventions may comprise safe and efficacious treatments.

## Author contributions

MS and AM created concept and design of the study, jointly analyzed the data, and wrote the manuscript; DR, HL, ML, SG, and MG provided the cardiological data and contributed to their analysis; FH provided the neuroradiological data; VS, HR, SP and UZ provided the neurological data and critically reviewed the manuscript.

### Conflict of interest statement

The authors declare that the research was conducted in the absence of any commercial or financial relationships that could be construed as a potential conflict of interest.
